# Amino-terminal proteolytic fragment of the axon growth inhibitor Nogo-A (Rtn4A) is upregulated by injury and promotes axon regeneration

**DOI:** 10.1016/j.jbc.2023.105232

**Published:** 2023-09-09

**Authors:** Yuichi Sekine, Xingxing Wang, Kazuna Kikkawa, Sachie Honda, Stephen M. Strittmatter

**Affiliations:** 1 Department of Neuroscience and Neurology, Cellular Neuroscience, Neurodegeneration & Repair Program, Yale School of Medicine, New Haven, Connecticut, USA; 2 Department of Cell Biology, Kyoto Pharmaceutical University, Kyoto, Japan

**Keywords:** axon, central nervous system (CNS), myelin, proteolysis, regeneration, outgrowth inhibition, cerebral cortex, trauma, spinal cord injury

## Abstract

After adult mammalian central nervous system injury, axon regeneration is extremely limited or absent, resulting in persistent neurological deficits. Axon regeneration failure is due in part to the presence of inhibitory proteins, including NogoA (Rtn4A), from which two inhibitory domains have been defined. When these inhibitory domains are deleted, but an amino-terminal domain is still expressed in a gene trap line, mice show axon regeneration and enhanced recovery from injury. In contrast, when there is no amino-terminal Nogo-A fragment in the setting of inhibitory domain deletion, then axon regeneration and recovery are indistinguishable from WT. These data indicated that an amino-terminal Nogo-A fragment derived from the gene trap might promote axon regeneration, but this had not been tested directly and production of this fragment without gene targeting was unclear. Here, we describe posttranslation production of an amino-terminal fragment of Nogo-A from the intact gene product. This fragment is created by proteolysis near amino acid G214-N215 and levels are enhanced by axotomy. Furthermore, this fragment promotes axon regeneration *in vitro* and acts cell autonomously in neurons, in contrast to the inhibitory extracellular action of other Nogo-A domains.Proteins interacting with the amino-terminal Nogo-A fragment by immunoprecipitation include HSPA8 (HSC70, HSP7C). Suppression of HSPA8 expression by shRNA decreases axon regeneration from cerebral cortical neurons and overexpression increases axon regeneration. Moreover, the amino-terminal Nogo-A fragment increases HSPA8 chaperone activity. These data provide an explanation for varied results in different gene-targeted Nogo-A mice, as well as revealing an axon regeneration promoting domain of Nogo-A.

Traumatic or ischemic injury of the adult brain or spinal cord frequently interrupts axonal connections with limited neuronal cell death. Axonal regeneration holds the potential to restore lost function, but generally fails in the adult mammalian central nervous system (CNS), such that persistent neurological deficits are common. The basis for failed axon regeneration is multifactorial, and includes the action of inhibitory extracellular molecules, as well as cell autonomous limits on neuronal growth and the absence of stimulatory factors (1). Amongst inhibitory molecules, the expression of Nogo-A (Rtn4A) by oligodendrocytes contributes to restricted axonal regeneration (2).

Studies of Nogo-A protein have revealed two inhibitory protein domains, a mid region Δ20 domain encoded by exon III unique to Nogo-A and not found in Nogo-B or Nogo-C splice forms, plus a C-terminal Nogo-66 domain encoded by exons common to all isoforms (Fig. S1*A*) (2, 3, 4, 5, 6). The action of the later is mediated by NgR1 (Rtn4R) and by PirB (LILRB3)-binding sites (4, 7, 8), while both ß1-integrin and S1PR2 receptors have been implicated in Δ20 action (3, 5, 9). The creation of gene-targeted mice raised the possibility that the amino-terminal domain of Nogo-A might also have the capacity to influence axon regeneration. A gene trap Rtn4A allele, *nogoA*^*trap*^*,* preserved expression of the first two exons of Nogo-A and inserted a truncating sequence in exon III to create a fragment consisting of the amino-terminal 309 aa of Nogo-A, but no expression of either Δ20 or Nogo-66 domain from the Nogo-A or Nogo-B transcripts (10, 11). In contrast, a deletion of the start codon for Nogo-A and Nogo-B, *nogoA*^*atg*^*,* eliminated the amino-terminal fragment as well as the inhibitory domains (11, 12). The phenotype of these two strains differed with detectable sprouting, regeneration, and recovery in the line maintaining the amino-terminal protein fragment expression, *nogoA*^*trap*^. One explanation of these findings is that the amino-terminal domain included in the first 309 aa of Nogo-A promotes neural repair. However, this activity was not tested directly, and the production of an amino-terminal fragment with such activity, apart from the inhibitory domains, was not assessed.

Our previous studies have shown that Nogo-A is subject to proteolysis by ßACE1 at a C-terminal site to release a 24 kDa fragment associated with exosomes (13). This exosome fraction has enhanced Nogo-A inhibitory activity, and its production is enhanced by spinal cord trauma. However, the processing of the remaining amino-terminal Nogo-A fragment has not previously been characterized in detail. Nogo-A may exhibit alternate membrane orientations in the endoplasmic reticulum, plasma membrane, and exosome with proteolytic processing occurring within the cytosol, the organelle lumen, or the extracellular space (Fig. S1*B*) (13, 14).

Here, we investigated the proteolytic fate of the Nogo-A amino-terminal protein and assessed its activity in axon regeneration assays. We observe proteolytic release of an amino-terminal fragment of about 213 aa and show that its production is regulated by CNS trauma. Moreover, this fragment promotes axon regeneration from cultured cortical neurons and interacts with HSPA8. These data reveal injury-regulated proteolytic release of a growth-promoting fragment of Nogo-A that distinguishes between Nogo-A gene–targeted lines and contributes to its biological function.

## Results

### Nogo-A is cleaved near G214-N215 and produces a 40 kDa amino-terminal fragment

**Figure 1:**
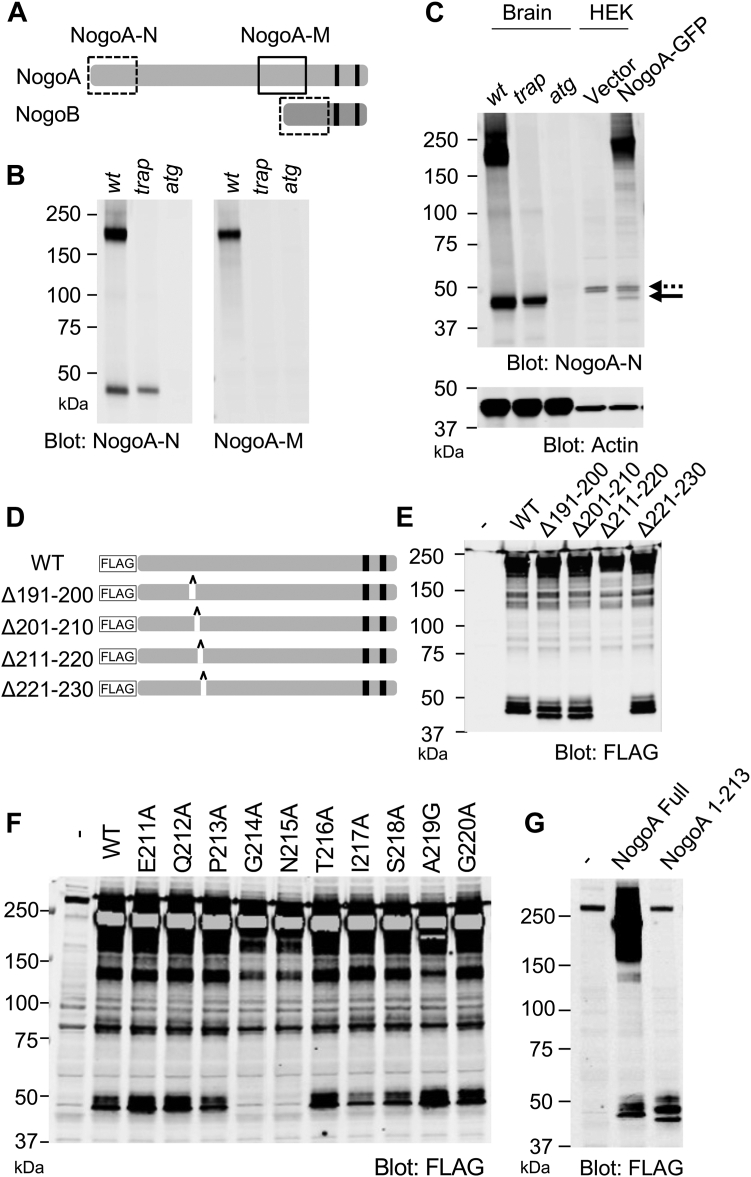
**Nogo-A amino-terminal is cleaved near G214 and N215.***A*, Nogo-A and Nogo-B proteins are shown as *filled rectangles*. Two transmembrane sites are indicated as *black bars*. Recognition sites of anti-NogoA-N antibody; *dashed-line square* and anti-NogoA-M antibody; *solid-line square* are shown. *B*, mouse brains from *wt*, *nogoA*^*trap/trap*^ or *nogoA*^*atg/atg*^ were lysed and immunoblotted with anti-NogoA-N or NogoA-M antibody. *C*, mouse brains from *wt*, *nogoA*^*trap/trap*^ or *nogoA*^*atg/atg*^, and HEK293T cells transfected with empty vector or C-terminal GFP-tagged Nogo-A (NogoA-GFP) were lysed and immunoblotted with anti-NogoA-N and anti-actin antibodies. *Dot arrow* shows nonspecific band and *solid arrow* shows Nogo-A fragment band. *D*, a series of FLAG-tagged Nogo-A 10 aa deletion constructs were generated. Schematical images of FLAG-tagged Nogo-A deletion mutants. *E*, HEK293T cells were transfected with WT or a series of deletion constructs of FLAG-Nogo-A. At 36 h after transfection, cells were lysed and immunoblotted with anti-FLAG antibody. *F*, HEK293T cells were transfected with WT or a series of point mutant constructs of FLAG-Nogo-A. At 36 h after transfection, cells were lysed and immunoblotted with anti-FLAG antibody. *G*, HEK293T cells were transfected with FLAG-Nogo-A WT or 1 to 213aa truncated mutant. At 36 h after transfection, cells were lysed and immunoblotted with anti-FLAG antibody. HEK, human embryonic kidney.

It has been predicted that the *nogoA*^*trap/trap*^ mutant mouse has the potential to generate a truncated form of Nogo-A protein terminated at aa 309 (11). To assess the presence of this truncated Nogo-A peptide in *nogoA*^*trap/trap*^ mouse brain, we used two different commercial antibodies, termed anti-NogoA-N (immunogen is rat Nogo-A aa 2–172) and anti-NogoA-M (immunogen is human Nogo-A aa 566–748). Based on the immunogen sequences, the anti-NogoA-N antibody detects both Nogo-A and Nogo-B proteins, while the anti-NogoA-M antibody detects only Nogo-A protein (Figs. 1*A* and S1*A*). Using these antibodies, we compared the expression of Nogo proteins in *wt*, *nogoA*^*trap/trap*^*,* and *nogoA*^*atg/atg*^ mouse forebrain lysate. Both anti-NogoA-N and NogoA-M antibodies detected the 200 kDa full-length Nogo-A in *wt* forebrain but not in either *nogoA*^*trap/trap*^ or *nogoA*^*atg/atg*^ samples (Fig. 1*B*). A 40 kDa band was detected by anti-NogoA-N antibody in *wt* lysate, and a similar size of band was observed in *nogoA*^*trap/trap*^ lysate, but not in *nogoA*^*atg/atg*^ lysate. One interpretation is that the 40 kDa band in *wt* is the Nogo-B isoform because predicted Nogo-B protein molecular weight from its amino acid sequence is around 38 kDa, but *nogoB* mRNA levels in brain are low (10).

As an alternative, we considered the possibility that the 40 kDa band in *wt* brain is a cleaved fragment of full-length Nogo-A (13). To evaluate the possibility that the 40 kDa band is cleaved Nogo-A fragment, lysate from C-terminal GFP–tagged Nogo-A (NogoA-GFP) overexpressed human embryonic kidney 293T (HEK293T) cells was immunoblotted with anti-NogoA-N antibody (Fig. 1*C*). Compared to vector transfection, Nogo-A–expressing lysate exhibited a 40 kDa immunoreactive protein similar in mobility to the material from *wt* and *nogoA*^*trap/trap*^ forebrain lysate. Further, we generated an N-terminal FLAG–tagged Nogo-A expression construct and checked the migration of the expressed protein by SDS-PAGE. An immunoblot with anti-FLAG antibody showed several fragment bands with a prominent band around 40 kDa (Fig. S2). These data suggest that the 40 kDa band detected by anti-NogoA-N antibody in *wt* and *nogoA*^*trap/trap*^ forebrain lysate is an N-terminal fragment of Nogo-A rather than Nogo-B.

To identify the cleavage site for the Nogo-A 40 kDa fragment, we created a series of ten amino acids deletion constructs (Fig. 1*D*). Deletion of ten amino acids in Nogo-A 191 to 200 (Δ191–200) and Δ201 to 210 constructs showed faster migration of Nogo-A N-terminal fragments than WT fragments, and Δ211 to 220 abolished the 40 kDa fragment (Fig. 1*E*). These results suggest the cleavage site is between 211 and 220 aa of NogoA. As a next step, we constructed a series of single aa substitution mutants from 211 to 220 aa and examined the migration pattern in SDS-PAGE. Substitution of Glycine at 214 with Alanine or Asparagine at 215 with Alanine abrogated the 40 kDa fragment (Fig. 1*F*). Furthermore, we generated a NogoA 1 to 213 aa truncated construct (termed NogoA-213) and compared its migration pattern with the Nogo-A N-terminal fragment cleaved from WT (Fig. 1*G*). NogoA-213–expressing HEK293T cells showed immunoreactive protein near 40 kDa, similar to the pattern for N-terminal fragments of WT Nogo-A. For the *nogoA*^*trap/trap*^ samples, the primary gene trap insertion is predicted to yield a primary translation product consisting of truncated Nogo-A aa 1 to 309, but this truncated Nogo-A as well as full-length Nogo-A appear to be cleaved near amino acid G214/N215 to yield a fragment similar to NogoA-213. The cleavage requires G214/N215 and produces a fragment of about 213 aa, but the cleaved peptide bond was not determined precisely. In subsequent studies we tested recombinant Nogo-A 1 to 213 functions.

### nogoA^trap/trap^ mouse brain has Nogo-A 40 kDa fragment but not Nogo-B

**Figure 2:**
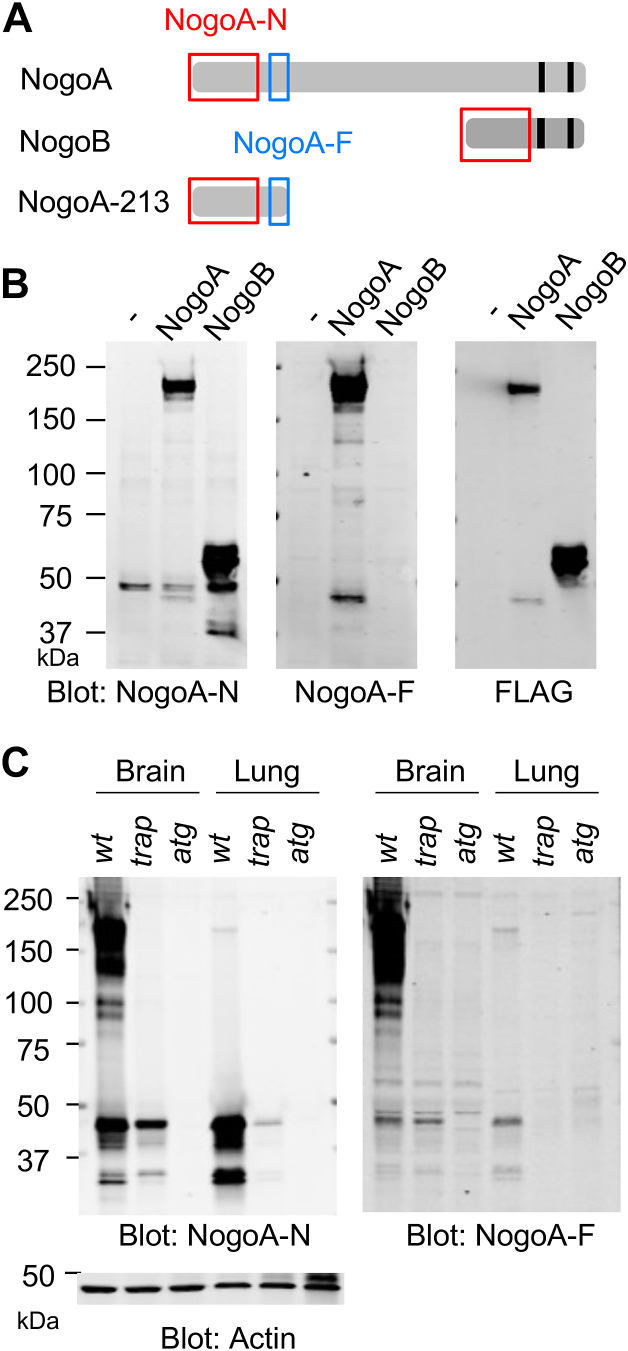
***nogoA***^***trap/trap***^**mouse brain has Nogo-A 40 kDa fragment but not Nogo-B.***A*, recognition sites of anti-NogoA-N antibody; *dashed square* and anti-NogoA-F antibody; *striped square* are shown. *B*, HEK293T cells were transfected with FLAF-tagged Nogo-A or FLAF-tagged Nogo-B. At 36 h after transfection, cells were lysed and immunoblotted with anti-NogoA-N, anti-NogoA-F, or anti-FLAG antibody. *C*, mouse brains and lungs from *wt*, *nogoA*^*trap/trap*^, or *nogoA*^*atg/atg*^ were lysed and immunoblotted with anti-NogoA-N, NogoA-M, NogoA-F, or actin antibody. HEK, human embryonic kidney.

The 186 to 213 aa sequence adjacent to the G214/N215 site required for cleavage is present in both full-length Nogo-A and its amino-terminal fragment, but not in Nogo-B (Fig. S3). Therefore, we produced a hNogo-A aa 186 to 213 specific antibody termed NogoA-F to distinguish between Nogo-A amino-terminal fragment and Nogo-B (Fig. 2*A*). To check the specificity of NogoA-F antibody, FLAG-tagged hNogo-A and hNogo-B constructs were transfected in HEK293T cells, and then cells were lysed and immunoblotted with anti-NogoA-N and NogoA-F antibodies (Fig. 2*B*). As expected, the anti-NogoA-N antibody detected full-length Nogo-A, NogoA-213 fragment, and Nogo-B. On the other hand, the anti-NogoA-F antibody detected full-length Nogo-A and NogoA-213, but not Nogo-B. Anti-FLAG antibody detected full-length Nogo-A at 200 kDa and the Nogo-A fragment at 40 kDa in Nogo-A–transfected lysate and Nogo-B around 60 kDa in Nogo-B–transfected lysate. Based on their molecular weights by SDS-PAGE, the Nogo-A N-terminal fragment and Nogo-B are clearly separable. These data validate the intended specificity of the NogoA-F antibody.

The NogoA-F antibody was employed for immunoblotting using mouse tissue lysate to monitor endogenous Nogo-A and Nogo-B expression, because the antigen sequence of human Nogo-A aa186 to 213 is 90% identical to mouse Nogo-A sequence (Fig. S3). Both anti-NogoA-N and NogoA-F antibodies detected full-length Nogo-A in the lysate from *wt* mouse brain but not in the *nogoA*^*trap/trap*^ and the *nogoA*^*atg/atg*^ brain (Fig. 2*C*). Notably, a specific band with identical migration at 40 kDa was detected in lysates from both *wt* and the *nogoA*^*trap/trap*^ mouse brains by both the anti-NogoA-N or anti-NogoA-F antibodies. This band was not present in *nogoA*^*atg/atg*^ brain. Thus, we conclude that the 40 kDa band in *wt* brain and *nogoA*^*trap/trap*^ brain is the Nogo-A N-fragment of aa 1 to 213, but not Nogo-B.

A 40 kDa Nogo protein species has been observed in *wt* lung samples using by anti-NogoA-N antibody. To confirm whether the 40 kDa band is Nogo-A N-fragment or Nogo-B, brain and lung lysate from *wt*, *nogoA*^*trap/trap*^ or *nogoA*^*atg/atg*^ animal were immunoprecipitated with anti-NogoA-N and anti-NogoA-F antibodies and immunoblotted with anti-NogoA-N antibody. Both NogoA-N and NogoA-F immunoprecipitates contained same size band around 40 kDa (Fig. S4). A very small amount of full-length Nogo-A was detected by anti-NogoA-N antibody in the lung lysates from *wt* animal, whereas the Nogo-A fragment was abundantly observed. These finding indicated that most of Nogo-A protein is proteolyzed to yield the NogoA-213 fragment in lung and little Nogo-B is present.

### Nogo-A amino-terminal fragment is increased after axotomy in cortical neuron culture

**Figure 3:**
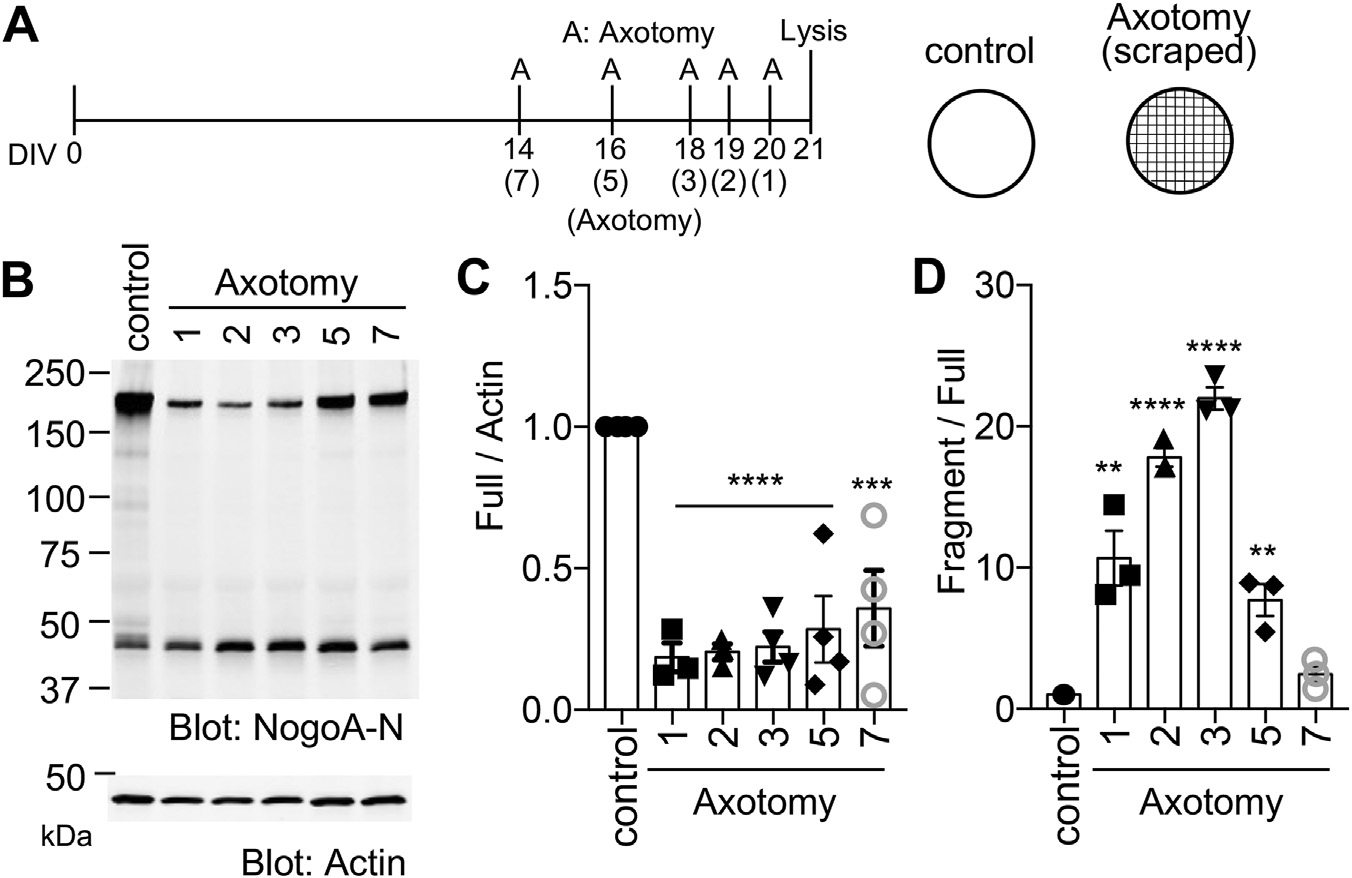
**Nogo-A amino-terminal fragment is increased after axotomy in cortical culture neurons.***A*, timeline for this experiment (*left*) and schematical illustration for axotomy (*right*). The axotomy was performed by pipet tip; the neurons were scraped by 10 × 10 times. *B*, neurons in 6-well plate were scraped at DIV 14, 16, 18, 19, 20, and lysed at DIV 21. The cell lysate was immunoblotted with anti-NogoA-N and anti-actin antibodies. *C*, the graph shows relative intensity value of full-length Nogo-A divided by actin. Mean ± SEM, N = 4 independent experiments. ∗∗∗*p* < 0.005, ∗∗∗∗*p* < 0.0001, one-way ANOVA followed by Dunnett's test (F = 13.84, DF = 21). *D*, the graph shows relative intensity value of full-length Nogo-A divided by Nogo-A fragment divided by full-length Nogo-A. Mean ± SEM, N = 3 independent experiments. ∗∗*p* < 0.01, ∗∗∗∗*p* < 0.005, ∗∗∗∗*p* < 0.0001, one-way ANOVA followed by Dunnett's test (F = 65.05, DF = 17). A; axotomy; DIV; day *in vitro*.

Since Nogo-A N-terminal cleaved fragment of aa 1 to 213 is detected in a brain tissue, we analyzed whether the fragment is regulated by neuronal injury. Mouse cortical neurons from embryonic day 17.5 (E17.5) were cultured for 2 weeks in a 6-well plate, and then the neurons were axotomized with a plastic tip at different time points and collected for immunoblotting (Fig. 3*A*). At 1 to 3 days after axotomy, the level of full-length Nogo-A protein was dramatically decreased, but then levels were restored by day 5 to 7 (Fig. 3, *B* and *C*). In contrast, the NogoA-213 fragment was increased after axotomy, relative to the full-length Nogo-A protein (Fig. 3, *B* and *D*). Thus, Nogo-A proteolysis is increased after axotomy, and the levels of the NogoA-213 fragment increases after injury of cultured cortical neurons.

### Nogo-A amino-terminal fragment is increased after spinal cord trauma *in vivo*

**Figure 4:**
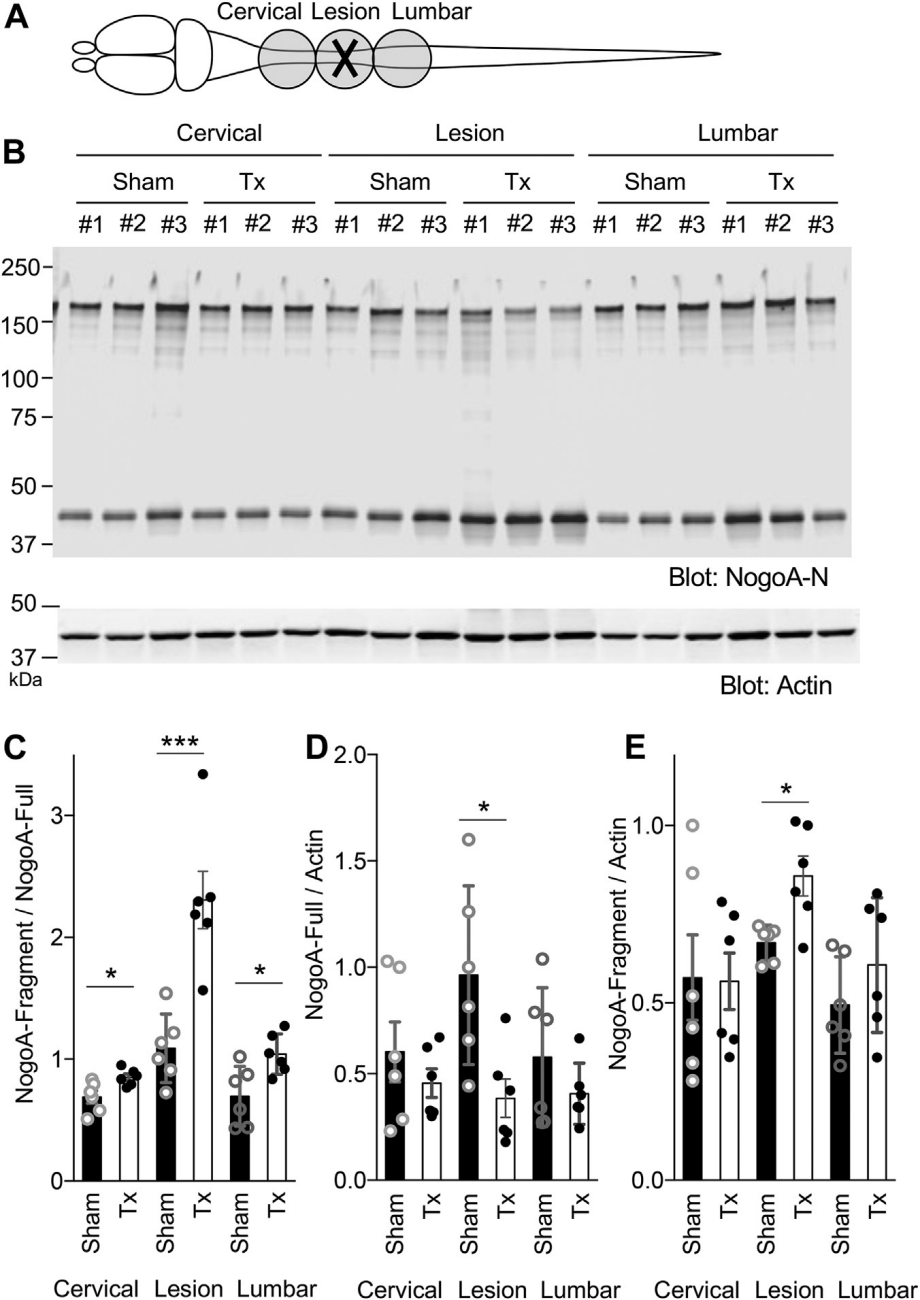
**Increased Nogo-A amino-fragment levels after spinal cord trauma *in vivo*.***A*, schematic image of collected spinal cord tissues for immunoblotting. *B*, spinal cord tissues 3 days after sham or transection surgery were lysed and immunoblotted with anti-NogoA-N and anti-actin antibodies. *C*, quantification of Nogo-A amino-fragment divided Nogo-A full-length intensity. Mean ± SEM, N = 6 animals. ∗∗∗*p* < 0.005, ∗*p* < 0.05, Student’s two-tailed *t* test. *D* and *E*, quantification of Nogo-A full length (*D*) or Nogo-A amino-fragment (*E*) divided actin intensity. Mean ± SEM, N = 6 animals. ∗*p* < 0.05, Student’s two-tailed *t* test.

The evidence that the N-terminal Nogo-A fragment was increased in cortical culture neuron after axotomy *in vitro*, raises the issue of whether the N-terminal Nogo-A fragment might be generated *in vivo* after neuronal injury. Production of a C-terminal Nogo-A fragment from a different cleavage site has been observed in tissue lysates after spinal cord injury in mice (13). To evaluate the relationship of NogoA-213 fragment production to trauma, mice were subjected to the spinal cord crush surgery. At 3 days postinjury, spinal cords from sham and injured (Tx) mice were collected and separated into three segments: the cervical area (rostral to the lesion), the lesion site, and the lumbar area (caudal to the lesion) (Fig. 4*A*). Tissues were lysed and immunoblotted with NogoA-N antibody with measurement of the intensity of Nogo-A full-length and amino-terminal NogoA-213 fragment signals (Fig. 4*B*). The ratio of NogoA-213 fragment to Nogo-A full length was significantly increased in injured tissues compared to sham surgery tissues (Fig. 4*C*, at the lesion site, *p* = 0.00091). For injured tissue samples, the level of Nogo-A full-length protein normalized to actin level was significantly decreased, and the level of NogoA-213 fragment to actin level was significantly increased (*p* = 0.0105) compared to the sham surgery tissues (Fig. 4, *D* and *E*). Thus, cleavage of Nogo-A to produce NogoA-213 fragment is increased after CNS trauma.

### Nogo-A amino-terminal fragment enhances axon regeneration in cortical neuron culture

Full-length Nogo-A is expressed in both oligodendrocytes and neurons, and its net effect is to inhibit axonal regeneration (2, 3, 4, 5, 6). Here, we assessed the functional effect of the injury-related production of the NogoA-213 fragment. To explore activity, we first produced and purified the NogoA-213 fragment as a recombinant protein from *Escherichia coli* (Fig. S5*A*). Cortical neurons collected at E17.5 and cultured for 8 days *in vitro* were axotomized with a metal pin tool and then incubated to allow regeneration in the presence of different doses of extracellular NogoA-213 fragment protein for 3 days (Fig. S5*B*). Regenerated axons in the scraped area were visualized by anti-ßIII-tubulin antibody, and the signal was measured to score axon regeneration (Fig. S6). The extracellular addition of recombinant NogoA-213 fragment had no discernable effect on the *in vitro* regeneration of cerebral cortical axons.

**Figure 5:**
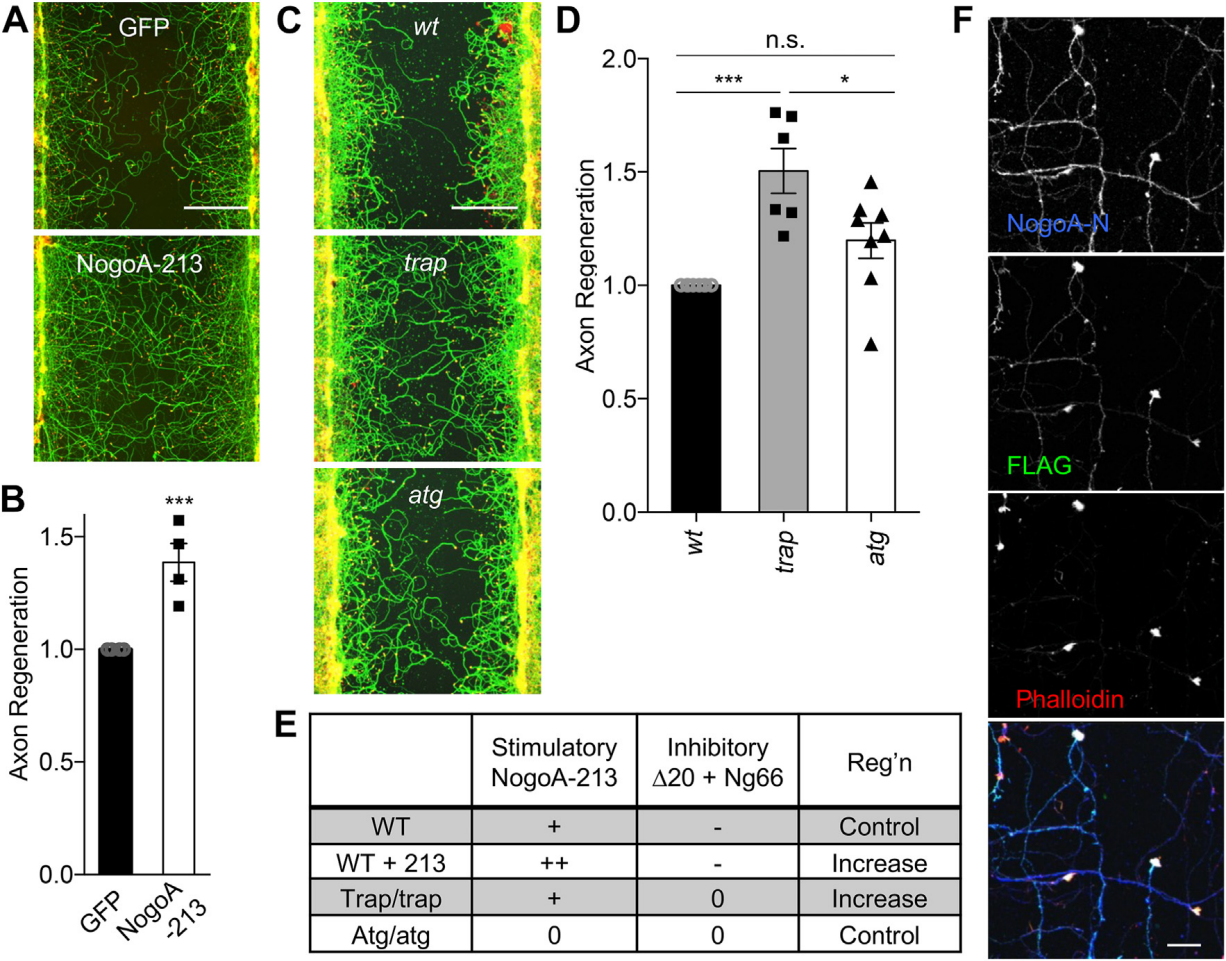
**Nogo-A amino-terminal fragment enhances axon regeneration in cortical culture neurons.***A*, representative images of regenerating axons in GFP and Nogo-A 1 to 213 transfected neurons 3 days after axotomy. The microphotographs show ßIII-tubulin (in axons; *green*) and phalloidin (to stain F-actin; *magenta*) to illustrate the growth cones of cortical neurons in the middle of the scraped area. The scale bar represents 200 μm. *B*, the graph shows quantification of axonal regeneration relative to GFP. Mean ± SEM, n = 4 biological replicates. ∗∗∗*p* < 0.005, Student’s two-tailed *t* test. *C*, representative images of regenerating axons in neurons from *wt*, *nogoA*^*trap/trap*^ or *nogoA*^*atg/atg*^ 3 days after axotomy. The microphotographs show ßIII-tubulin (in axons; *green*) and phalloidin (to stain F-actin; *magenta*) to illustrate the growth cones of cortical neurons in the middle of the scraped area. The scale bar represents 200 μm. *D*, the graph shows quantification of axonal regeneration relative to *wt*. Mean ± SEM, n = 5 to 8 biological replicates. n.s., not significant, (*p* > 0.05), ∗*p* < 0.05, ∗∗∗*p* < 0.005, one-way ANOVA followed by Tukey's test (F = 13.13, DF = 21). *E*, the table summarizes the expression of NogoA domains and the degree of axon regeneration. *F*, localization of Nogo-A in regenerating neurons. Cortical neurons were transfected with FLAG-NogoA 1 to 213. Neurons were scraped at DIV 8 and regenerated for 3 days. Confocal microscope images of FLAG-NogoA 1 to 213 (FLAG; *green*), NogoA full or N-fragment (NogoA-N; *blue*), and growth cones (rhodamine-phalloidin; *red*) are taken. The scale bar represents 20 μm.

While extracellular NogoA-213 did not alter regeneration, we considered whether intracellular neuronal NogoA-213 fragment might modify axonal regeneration. Intracellular action is suggested by previous Nogo-A protein localization studies. Although a subset of Nogo-A has been detected on the surface of oligodendrocytes where it might be released into the extracellular space, in other cell types most of the protein is associated with endoplasmic reticulum and the amino terminus is cytoplasmic (2, 7, 15). To assess intracellular cell-autonomous NogoA-213 action, we transfected cortical neurons with a vector-expressing FLAG–tagged NogoA-213, then cultured for 8 days, then axotomized with a metal pin tool, and finally incubated for further 3 days to assess axon regeneration (Fig. 5, *A* and *B*). The expression of NogoA-213 or control GFP was confirmed by immunoblotting (Fig. S7). Regenerated axons in the scraped area were visualized by anti-ßIII-tubulin antibody and quantified. NogoA-213 fragment overexpression yielded a 1.4-fold increase in axonal regeneration relative to GFP control (*p* = 0.0038).

Since overexpressed NogoA-213 has stimulatory effect for axonal regeneration, we sought to evaluate the effect of endogenous NogoA-213 on regeneration. Neurons from *wt*, *nogoA*^*trap/trap*^*,* or *nogoA*^*atg/atg*^ animals were subjected to *in vitro* axonal regeneration assay under our standard conditions with very few glia and no myelin (Fig. 5*C*). Axon regeneration by neurons from *nogoA*^*trap/trap*^ mice was significantly enhanced compared to *wt* and *nogoA*^*atg/atg*^ neurons (Fig. 5, *D* and *E*). Since the specific difference between *nogoA*^*trap/trap*^ or *nogoA*^*atg/atg*^ neurons is NogoA-213 expression, we conclude that NogoA-213 promotes axonal regeneration. The WT condition involves the expression of both regeneration promoting and inhibiting domains of NogoA (Fig. 5*E*).

Although full-length Nogo-A associates with endoplasmic reticulum and plasma membrane *via* two hydrophobic segments near the carboxyl terminus, the NogoA F-fragment lacks predicted transmembrane segments. To elucidate NogoA-213 fragment localization in injured neurons, FLAG-tagged NogoA-213–transfected cortical neurons were stained with anti-FLAG and anti-NogoA-N antibodies 3 days after axotomy (Fig. 5*F*). The FLAG-based signal for NogoA-213 was highly enriched in regenerating growth cones, which were costained for F-actin with rhodamine-conjugated phalloidin. Relatively little NogoA-213 was detected along the length of the axon shaft. The anti-NogoA-N signal, reflecting both NogoA-213 and endogenous full-length Nogo-A, was found in the axon shafts and the growth cones. Thus, the cleaved NogoA-213 fragment localizes to regenerating growth cone, where it acts intracellularly to enhance axonal regeneration.

### Nogo-A amino-terminal fragment interacts with HSPA8

**Figure 6:**
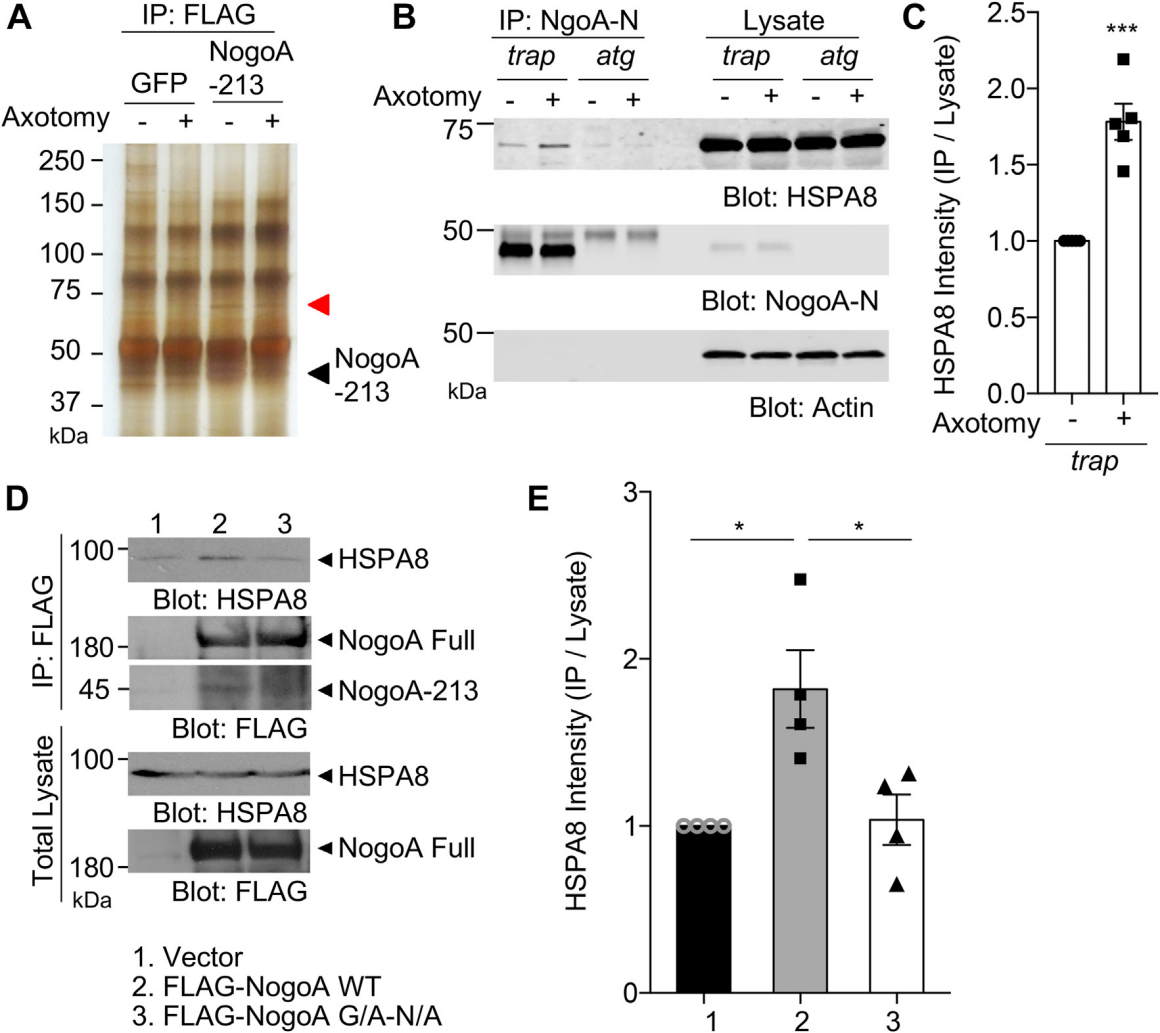
**Nogo-A amino-terminal fragment interacts with HSPA8.***A*, cortical neurons transfected with GFP or FLAG-NogoA 1 to 213 were without or with axotomy for 16 h and then the lysate was immunoprecipitated with anti-FLAG antibody. The immunoprecipitates were resolved by SDS-PAGE and visualized by silver staining. *Red arrow band* was excised and analyzed by LC/MS to determine its identity. *Black arrow* shows immunoprecipitated NogoA 1 to 213 protein band. *B*, cortical culture neurons of *nogoA*^*trap/trap*^ or *nogoA*^*atg/atg*^ without or with axotomized for 16 h were lysed and immunoprecipitated with anti-NogoA-N antibody. The immunoprecipitates were resolved by SDS-PAGE and immunoblotted with anti-HSPA8, anti-NogoA-N, and anti-actin antibody. *C*, quantification of HSPA8 protein levels in the immunoprecipitates normalized to total cell lysate. Mean ± SEM, n = 5 biological replicates. ∗∗∗*p* < 0.005, Student’s two-tailed *t* test. *D*, HEK293T cells were transfected with FLAG-tagged NogoA WT or FLAG-tagged NogoA G214A-N215A mutant. At 36 h after transfection, cells were lysed and immunoprecipitated with anti-FLAG antibody. Then, the immunoprecipitates were resolved by SDS-PAGE and immunoblotted with anti-HSPA8 and anti-FLAG antibodies. *E*, quantification of HSPA8 protein levels in the immunoprecipitates normalized to total cell lysate. Mean ± SEM, n = 3 biological replicates. ∗*p* < 0.05, one-way ANOVA followed by Tukey's test (F = 9.66, DF = 8). HEK, human embryonic kidney.

To explore mechanisms whereby the NogoA-213 fragment enhances axonal regeneration in neurons, we sought to identify proteins that form a physical complex with NogoA-213. Control GFP or FLAG-tagged NogoA-213–expressing cortical neurons with or without axotomy were lysed and immunoprecipitated with anti-FLAG antibody. The immunoprecipitates were resolved by SDS-PAGE and visualized by silver staining (Fig. 6*A*). FLAG–NogoA-213 complexes contained a single sharp protein band around 70 kDa in both intact and axotomized neurons. The band was excised, digested with trypsin, and peptides were analyzed by LC/MS to determine the identity. From the analysis, we identified HSPA8 (heat shock protein family [HSP70] member 8; HSC70, heat shock cognate 70 kDa protein; HSP7C) as a prominent binding partner of NogoA-213 fragment in axotomized cortical neurons. To confirm this binding, cortical neurons from *nogoA*^*trap/trap*^ or *nogoA*^*atg/atg*^ mice were cultured and axotomized, then immunoprecipitated with anti-NogoA-N antibody and immunoblotted with anti-HSPA8 antibody (Fig. 6*B*). The interaction between endogenous NogoA N-fragment and HSPA8 was observed in *nogoA*^*trap/trap*^ neurons, but not in *nogoA*^*atg/atg*^ neurons, and the level of the protein complex intensity was significantly increased after axotomy (Fig. 6*C*). We conclude that HSPA8 is the physical interactor of NogoA-213 in regenerating axons.

We compared the HSPA8 interaction of Nogo proteins from cells expressing NogoA WT *versus* a NogoA G214A-N215A mutant, which lacks the proteolytic processing sites to produce NogoA-213. Lysates from HEK293T cells expressing FLAG-tagged NogoA WT or NogoA G214A-N215A were subjected to immunoprecipitation with anti-FLAG. Despite equal amounts of full-length Nogo-A expression, NogoA-213 was present selectively in the NogoA WT cells. The coimmunoprecipitation of endogenous HSPA8 was detected above background for NogoA WT transfected HEK293T cells, but not for NogoA G214A-N215A cells, suggesting that HSPA8 interacts preferentially with the NogoA-213 fragment as compared to unprocessed full length of NogoA (Fig. 6, *D* and *E*).

### HSPA8 regulates axonal regeneration *in vitro*

**Figure 7:**
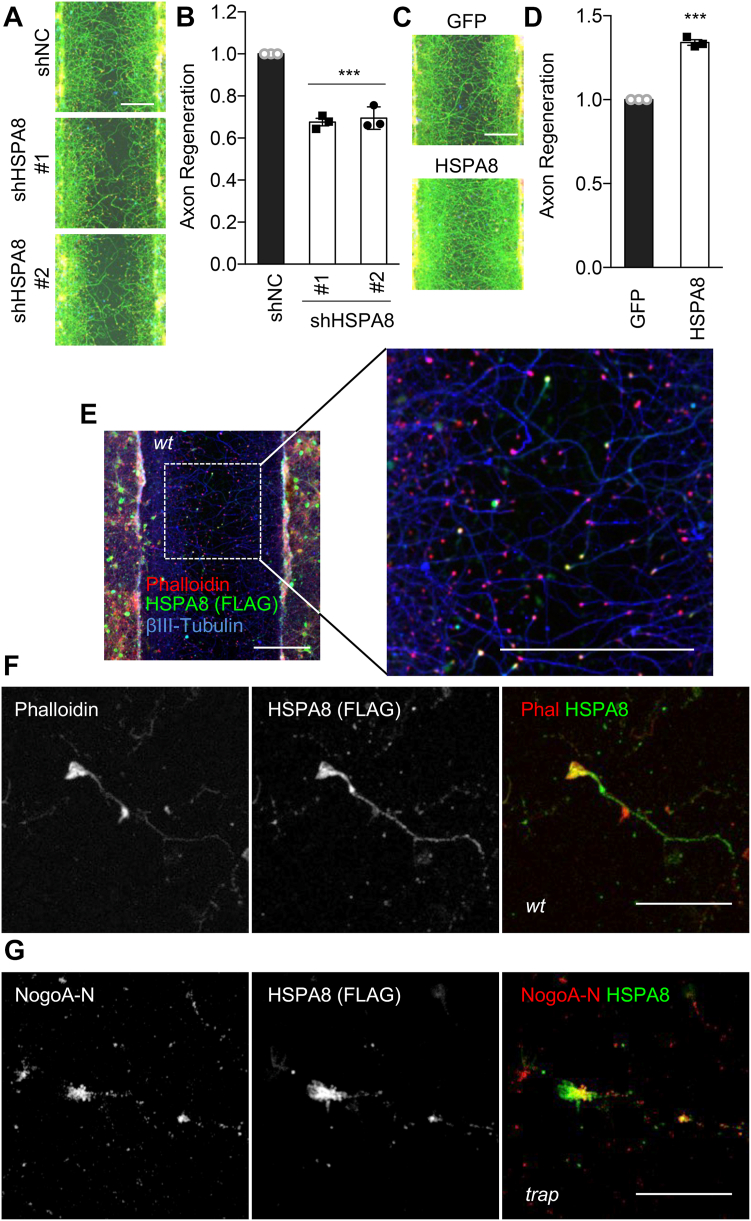
**HSPA8 regulates axonal regeneration *in vitro*.***A*, representative images of regenerating axons in shNC, shHSPA8 #1, and #2 transfected neurons 3 days after axotomy. The microphotographs show ßIII-tubulin (in axons; *green*) and phalloidin (to stain F-actin; *magenta*). The scale bar represents 200 μm. *B*, quantification of axonal regeneration relative to shNC. Mean ± SEM, n = 3 biological replicates. ∗∗∗*p* < 0.005, one-way ANOVA followed by Dunnett's multiple comparisons test (F = 77.83, DF = 8). *C*, representative images of regenerating axons in GFP or HSPA8 transfected neurons 3 days after axotomy. The scale bar represents 200 μm. *D*, quantification of axonal regeneration relative to GFP. Mean ± SEM, n = 3 biological replicates. ∗∗∗*p* < 0.005, Student’s two-tailed *t* test. *E*, cortical neurons taken from *wt* mouse were transfected with FLAG-HSPA8 and scraped at DIV 8 and regenerated for 3 days. Low magnification image of FLAG-HSPA8 (anti-FLAG antibody; *green*), axons (anti-ßIII-tubulin antibody; *blue*), and growth cones (rhodamine-phalloidin; *red*) are taken. The scale bar represents 200 μm in both panels. *F*, cortical neurons taken from *wt* mouse were transfected with FLAG-HSPA8 and scraped at DIV 8 and regenerated for 3 days. Confocal microscope images of FLAG-HSPA8 (anti-FLAG antibody; *green*), and growth cones (rhodamine-phalloidin; *red*) in scraped area are taken. The scale bar represents 20 μm. *G*, cortical neurons taken from *nogoA*^*trap/trap*^ mouse were transfected with FLAG-HSPA8 and scraped at DIV 8 and regenerated for 3 days. Confocal microscope images of FLAG-HSPA8 (anti-FLAG antibody; *green*), Nogo-A amino-terminal fragment (anti-NogoA-N antibody; *blue*), and growth cones (rhodamine-phalloidin; *red*) in scraped area are taken. The scale bar represents 20 μm.

There is no report demonstrating a functional role for HSPA8 in axonal regeneration. Recently, we reported a genome-wide loss-of-function screen for factors limiting axonal regeneration using lentiviral shRNA library with scrape axotomy *in vitro* cortical culture neurons (16). In the screen, we evaluated about 17,000 mouse genes and identified about 100 genes, whose suppression decreases axonal regeneration. Notably, HSPA8 was in the top 20 out of the 100 genes from this screen. More focused functional studies for HSPA8 have not been conducted previously. Here, we assessed the effect of HSPA8 expression on axonal regeneration in cortical culture neurons. First, we generated two different shRNA knockdown constructs (#1 and #2) targeting HSPA8 and examined axonal regeneration from cortical neurons. Knockdown of HSPA8 resulted in significant suppression of axonal regeneration compared with nontargeting shRNA control (Fig. 7, *A* and *B*). Each of shHSPA8 #1 and #2 constructs significantly decreased the HSPA8 protein expression in cortical culture neurons (Fig. S8, *A* and *B*). In contrast, neurons transfected with an HSPA8 expression vector showed enhanced axonal regeneration compared with the GFP control (Figs. 7, *C* and *D* and S8, *C* and *D*).

**Figure 8:**
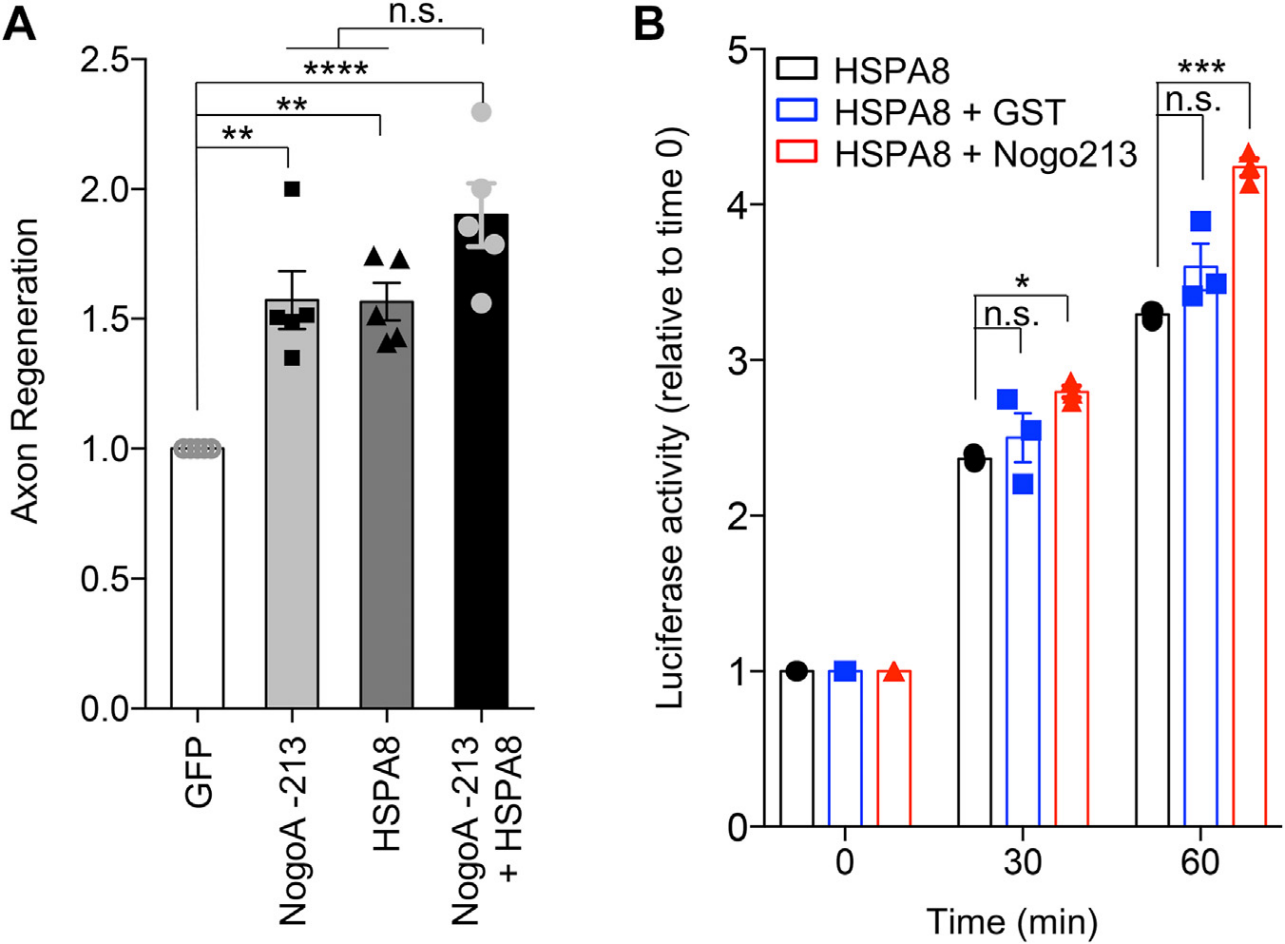
**NogoA N-terminal fragment enhances HSPA8 refolding activity.***A*, quantification of axonal regeneration of GFP, NogoA-213, HSPA8, and NogoA-213+HSPA8 transfected neurons relative to GFP. Mean ± SEM, n = 4 biological replicates. n.s., not significant (*p* > 0.05), ∗∗*p* < 0.01, ∗∗∗∗*p* < 0.001, one-way ANOVA followed by Tukey's test (F = 17.23, DF = 19). *B*, recombinant HSPA8 protein was incubated with GST or NogoA-213 at 37 °C for 15 min. Denatured luciferase mixed with HSPA8/GST or HSPA8/NogoA-213 was incubated at 30 °C for the indicated time points. To determine the luciferase activity, samples were added to luciferin and the luminescence was measured. The graph sows fold change. Mean ± SEM, n = 3 replicates. ∗*p* < 0.05, ∗∗∗*p* < 0.005, one-way ANOVA followed by Dunnett's test (30 min, F = 5.44, DF = 8, 60 min, F = 26.89, DF = 8). HSP, heat shock protein.

Since NogoA-213 localizes at growth cone and associates with HSPA8, we hypothesized that HSPA8 would localize to regenerating growth cones. We observed HSPA8 enrichment within regenerating growth cones of *wt* neurons (Fig. 7, *E* and *F*) and colocalization of HSPA8 with Nogo-A N immunoreactivity in *nogoA*^*trap/trap*^ neurons (Fig. 7*G*). Based on colocalization and similar action, we considered whether NogoA-213 and HSPA8 overexpression might have synergistic effects on axon regeneration. Neurons transfected with GFP, NogoA-213, HSPA8, or NogoA-213+HSPA8 were assessed for regeneration assay (Fig. S9). While dual overexpression of Nogo-213 and HSPA8 significantly enhanced axonal regeneration compared to GFP control, the enhancement was not significantly greater than with Nogo-213 or HSPA8 single overexpression (Fig. 8*A*).

### Nogo-A amino-terminal fragment enhances HSPA8 refolding activity

The data above do not reveal whether HSPA8 regulates NogoA-213 or vice versa. We evaluated potential functional effects of the NogoA-213 fragment on HSPA8 enzymatic activity by measuring HSPA8-mediated protein refolding *in vitro*. HSPA8-mediated refolding of heat-denatured luciferase was significantly enhanced upon coincubation with NogoA-213 protein but not with GST control (Fig. 8*B*).

## Discussion

The major finding of the current study is the regulated proteolytic release of an amino-terminal fragment of Nogo-A with axon regeneration promoting activity. Use of a domain selective antibody, epitope tagging, and point mutations demonstrated that cleavage requires G214/N215 and produces a fragment similar to NogoA-213. Axotomy increased the level of this fragment in culture and in the spinal cord. Overexpression of this isolated fragment increased axon regeneration from cerebral cortical neurons. While comparison of a gene trap mouse mutant line with a null allele had suggested that an activity might be created (10, 11, 12), the data here demonstrate this growth promoting activity is generated from the WT allele, providing a likely explanation for the previous spinal cord injury outcomes. The activity documents a potential mechanism for greater neural repair in the gene trap line and implicates HSPA8 chaperone activity in the mechanism.

While we have mapped the site of Nogo-A cleavage near aa G214/N215, the nature of the responsible protease(s) has(have) not been determined here. Future studies will be required to define the responsible enzymes. It is known that calpains are activated after axotomy, so they are potential candidates (17, 18). Calpain cleavage site specificity for other substrates has been reported to depend on residues near but not necessarily at the cleaved peptide bond (19). Of note, we have previously shown that apart from this amino-terminal cleavage, a carboxyl fragment of Nogo-A is released into exosomes by ßACE1 action (13).

The two inhibitory Nogo-A domains can function independently of one another through cell surface receptors. Different receptor mechanisms have been implicated for these distinct domains. For the mid region Δ20 domain, there are cellular actions *via* integrins on multiple cell types (3) and additional actions *via* S1PR2 receptors (9). For the Nogo-66 domain acting through either NgR1 or LilRB2 (7, 8) release into exosomes and the presence of surrounding amino acid sequence enhances affinity to pM levels (4, 13). Several coreceptors for Nogo-66 action *via* NgR1 have been described, including PlexinA2 (20). Additional inhibitors of axon regeneration, MAG and OMgp, also bind with high affinity to NgR1 and LilRB2 (4, 8, 21, 22).

In contrast to these receptor-mediated inhibitory responses of extracellular Nogo-A, the amino-terminal proteolytic fragment is likely to act cell autonomously as a cytoplasmic fragment. Expression of this fragment yields a soluble cytoplasmic fragment and NogoA-213–expressing neurons show increased regeneration. Moreover, extracellular recombinant amino-terminal fragment had no effect on regeneration. The amino acid composition of the amino-terminal 1 to 213 fragment is atypical and highly enriched for proline and for glutamate. This provides the likely explanation for the aberrantly slow migration by SDS-PAGE.

Assessment of protein coimmunoprecipitation with the Nogo-A amino-terminal fragment revealed an interaction with HSPA8, and alterations of HSPA8 expression were positively correlated with the extent of axon regeneration from cortical neurons. Other heat shock proteins, such as HSP27, is known to be regulated by axotomy and when overexpressed to support axon regeneration (23, 24, 25, 26), though only HSPA8 was detected here. The association of NogoA with with Apg-1 (HSPA4L, Osp94) of the HSP110 family has been described previously in one report (27). HSPA8 itself is upregulated in the injured spinal cord and plays a role in titrating the extent of ischemic damage (28).

Here, the presence of NogoA-213 increased HSPA8 chaperone activity in a biochemical assay with luciferase as a substrate. These data raise the possibility that the NogoA-213 fragment within regenerating axonal growth cones may have a novel function to facilitate HSPA8 chaperone activity and thereby increase axon extension. Regulation of chaperone activity might provide a parallel mechanism for amino-terminal Nogo-A fragment to increase axon regeneration.

In conclusion, we demonstrate the increased production of a proteolytic fragment of Nogo-A with axon regeneration promoting effects. Its action is distinct from previously described Nogo-A inhibitory domains. A gene trap mutant allele of Nogo-A preserves this sequence while the inhibitory Nogo-A domains and exhibits the more robust axonal regeneration phenotype.

## Experimental procedures

### Vertebrate animals

All procedures and postoperative care were performed with approval of the Yale University Institutional Animal Use and Care Committee. The *nogoA*^*trap/trap*^ and *nogoA*^*atg/atg*^ mouse lines have been described (10, 12) and were backcrossed for more than ten generations to C57BL/6 WT mice.

### Cell culture and transfection

HEK293T was maintained in DMEM containing 10% fetal bovine serum, 100 U/ml penicillin, and 100 μg/ml streptomycin. HEK293T cells were transfected using Lipofectamine 2000 (Invitrogen) following manufacturer’s instruction.

Mouse E17.5 cortical neurons were dissected in Hibernate E medium (catalog #HE-Ca; BrainBits) and incubated in digestion Hanks' balanced salt solution containing 30 U/ml Papain (catalog #LS003127; Worthington Biochemical), 2.5 mM EDTA, and 2 mg/ml DNaseI (catalog #DN25; Sigma) at 37 °C for 20 min. Digested tissues were triturated and suspended in Neurobasal-A media supplemented with B-27, GlutaMAX, and penicillin-streptomycin (all from Invitrogen). Neurons were plated on tissue culture plates coated with poly-d-lysine. Cortical neurons were transfected using Amaxa mouse neuron nucleofector kit according to the manufacture’s protocol. Before to plate, the dissected 4 × 10^6^ cortical neurons were resuspended in 100 μl of nucleofector solution and mixed with 2.5 μg of plasmid. Then, neurons were nucleofected using the program O-005 and plated on poly-D-lysine-coated 96-well plate for regeneration assay, 6-well plate for immunoprecipitation, 24-well plate for Western blot, or 8-well glass chamber slide for confocal imaging.

### Expression plasmids, antibodies, and reagents

C-terminal–tagged WT human Nogo-A plasmid has been previously described (2) and used for generating N-terminal FLAG–tagged Nogo-A and mutant constructs by PCR-methods using KOD Hot start DNA polymerase (TOYOBO) and sequenced. HSPA8 expression plasmid (#OHu27538D) purchased from GenScript was subcloned to pAAV-CAG vector. pAAV-U6-GFP expression vector (#VPK-413, Cell Biolabs) was used for making shRNA constructs. Targeting shRNA sequences are shNC:TTCTCCGAACGTGTCACGT, shHSPA8#1: GCTCGATTTGAGGAGTTGAAT, and shHSPA8#2: CGTAGGTTTGATGATGCTGTT. Anti-Myc (#M4439 or #C3956), anti-FLAG (#F7425), and anti-beta-actin (#A1978) antibodies (Sigma), anti-ßIII-tubulin antibody (#G7121, Promega), anti-Nogo-A (#AF3098 or #AF3515, R&D), anti-HSPA8 (#D12F2, Cell Signaling Technology), and anti-GFP antibody (#sc-9996, Santa Cruz Biotechnology) were used for following experiments. Rabbit polyclonal antibody against Nogo-A aa186 to 213 was generated using purified GST-fused Nogo-A 186 to 213 as an antigen (Covance). Rhodamine phalloidin (#R415) was purchased from Thermo Fisher Scientific.

### Immunoprecipitation, immunoblotting, silver staining, and mass spectrometry

Cortical culture neurons were lysed with a radioimmunoprecipitation assay buffer (50 mM Tris–HCl pH7.4, 150 mM NaCl, 1 mM EDTA, 0.1% SDS, 0.5% sodium deoxycholate and 1% Triton X-100). Mouse tissues were dissected and sonicated in radioimmunoprecipitation assay buffer. The lysate was centrifuged at 20,000*g* for 20 min at 4 °C. The supernatants were added with the antibody and protein G-sepharose mixture and incubated for 2 h at 4 °C with gentle rotation. The beads were washed three times, and the immune complexes were then resolved by SDS-PAGE. After transfer, nitrocellulose membranes were incubated in blocking buffer (Blocking Buffer for Fluorescent Western Blotting, Rockland MB-070-010) for 1 h at room temperature and immunoblotted with the appropriate primary antibodies. Following primary antibody incubation, secondary antibodies (Odyssey IRDye 680 or 800) were applied for 1 h at room temperature. Membranes were then washed and visualized using a Licor Odyssey Infrared imaging system. For mass spectrometry (MS), the gel was stained by Silver Stain MS kit (Wako, #299-58901) according to manufacturer’s instruction, and the bands were excised and subjected to analysis by MS (the MS and Proteomics Resource of the WM Keck Foundation Biotechnology Resource Laboratory at Yale University).

### Cortical axon regeneration assay

Primary neuron cultures were obtained from WT, *nogoA*^*trap/trap*^ and *nogoA*^*atg/atg*^ mice. Regeneration assay was performed as described previously (20). Primary cortical cultures were established from E17 C57BL/6 mice. Digested cells were plated on 96-well poly-d-lysine–coated plates at a density of 25,000 cells per well in 200 μl of plating medium. On 8 div, 96-well cultures were scraped using a custom-fabricated 96-pin array and the indicated amount of Nogo22 or exosomes were added. Neurons were allowed to regenerate for another 72 h before fixing with 4% paraformaldehyde. Regenerating axons in the scrape zone were visualized using an antibody against βIII tubulin (1:2000). Growth cones were visualized by staining for F-actin using rhodamine-conjugated phalloidin (1:2000, #R415, Life Technologies). Images were taken on a 10× objective in an automated high-throughput imager (ImageXpress Micro XLS, Molecular Devices) under identical conditions. Regeneration zone identification, image thresholding, skeletonizing, and quantitation were performed in Image J (https://imagej.nih.gov/ij/) to score axon regeneration extent. Measurements from different wells for the same condition in any one experiment were averaged together for one n value, and statistics were calculated between cultures from n embryos.

### Immunocytochemistry

At DIV 8 neurons were scraped with pipet tip and allowed for 3 days to regenerate. Then, cells were fixed with 4% paraformaldehyde for 15 min, and then permeabilized with 0.1% Triton X-100 in PBS for 15 min. Regenerating axons in the scrape zone were visualized using an antibody against βIII tubulin (1:2000, mouse monoclonal; catalog #G712A; Promega). FLAG-NogoA-213 or FLAG-HSPA8 were stained with anti-FLAG (1:1000, Sigma-Aldrich, #F7425) and endogenous Nogo-A was stained with anti-Nogo-A antibodies. Then, either Alexa-488–conjugated donkey anti-rabbit immunoglobulin G and Alexa-647–conjugated donkey anti-goat immunoglobulin G (1:2000, all from Invitrogen) were used to detect primary antibodies. Growth cones were visualized by staining for F-actin using rhodamine-conjugated phalloidin (1:2000, catalog #R415, Life Technologies). Samples were mounted with mounting solution (Vector Laboratories) and observed using a LSM710 confocal microscope. Obtained pictures were analyzed using Image J.

### Spinal cord injury

WT female mice were subjected to spinal cord crush surgery as described previously (29, 30). Animals received subcutaneous injection of buprenex (0.01 mg/kg) 30 min before surgery and were deeply anesthetized with ketamine (100 mg/kg) and xylazine (15 mg/kg). To expose the dorsal spinal cord at T7 and T8 levels, laminectomy was performed. Then spinal cord was fully crushed with forceps for 3 s. Forceps (Dumont No.5) had been filed to a width of 0.2 mm tips. The tips were inserted to include whole spinal cord across the ventral bone to avoid any spare tissue ventrally and laterally. Sham animals were laminectomized but not crushed. Muscle and skin overlying the lesion were sutured. Animals received subcutaneous injections of 100 mg/kg ampicillin and 0.1 mg/kg buprenex twice a day for the first 2 days after surgery. Three days after injury, animals were sacrificed, and spinal cords were taken to homogenize with radioimmunoprecipitation assay and sonicated for Western blot samples.

### *In vitro* refolding assay

*In vitro* luciferase refolding assay as described previously (31) was adapted for measuring HSPA8 refolding activity. Briefly, recombinant HSPA8 protein (400 ng) (catalog #NBP1-30278; Novus) was incubated with GST or NogoA-213 (400 ng) at 37 °C for 15 min. Firefly luciferase (2 × 10^8^ U) (catalog #SRE0045; Merck) in refolding buffer (25 mM Tris–HCl pH7.6, 50 mM KCl, 10 mM MgCl_2_, 5 mM DTT, 2.5 mM ATP) was heat denatured at 45 °C for 7 min. Denatured luciferase was mixed with HSPA8/GST or HSPA8/NogoA-213 and incubated on ice for 10 min, then the mixture was incubated at 30 °C for the indicated time points. To determine the luciferase activity, 2 μl sample was transferred to 20 μl luciferin (1 mM) (catalog #L9504; Merck) and luminescence was measured by luminometer (infinite M200, TECAN).

### Statistics

Statistical comparisons included one-way ANOVA and Student’s *t* test as specified in the Figure legends using Excel (https://www.microsoft.com/en-us/microsoft-365/excel) and Prism software (http://www.graphpad.com/). Statistical significance was set at *p* <0.05. All data are mean ± SEM. No statistical methods were used to calculate sample size estimates.

## Data availability

All data are contained within the manuscript.

## Supporting information

This article contains supporting information (2, 3, 13, 14, 32, 33).

## Conflict of interest

S. M. S. is a founder of, is a consultant for, and holds equity interest in ReNetX Bio, Inc, as well as being an Inventor on NgR1 (Rtn4R) intellectual property licensed from Yale to ReNetX Bio. The other authors declare that they have no conflicts of interest with the contents of this article.
