# Supplementary material for: Amino-terminal proteolytic fragment of the axon growth inhibitor Nogo-A (Rtn4A) is upregulated by injury and promotes axon regeneration
Source: J Biol Chem. 2023 Sep 9;299(10):105232. doi: 10.1016/j.jbc.2023.105232 (PMC10622843; doi:10.1016/j.jbc.2023.105232)
Supplement: Supporting Figures S1–S9 [file mmc1.pdf]

SUPPLEMENTARY INFORMATION

Amino-terminal proteolytic fragment of the axon growth inhibitor Nogo-A (Rtn4A) is upregulated by injury and promotes axon regeneration

Yuichi Sekine, Xingxing Wang, Kazuna Kikkawa, Sachie Honda, and Stephen M. Strittmatter

Contains Supplementary Figures S1-S9.

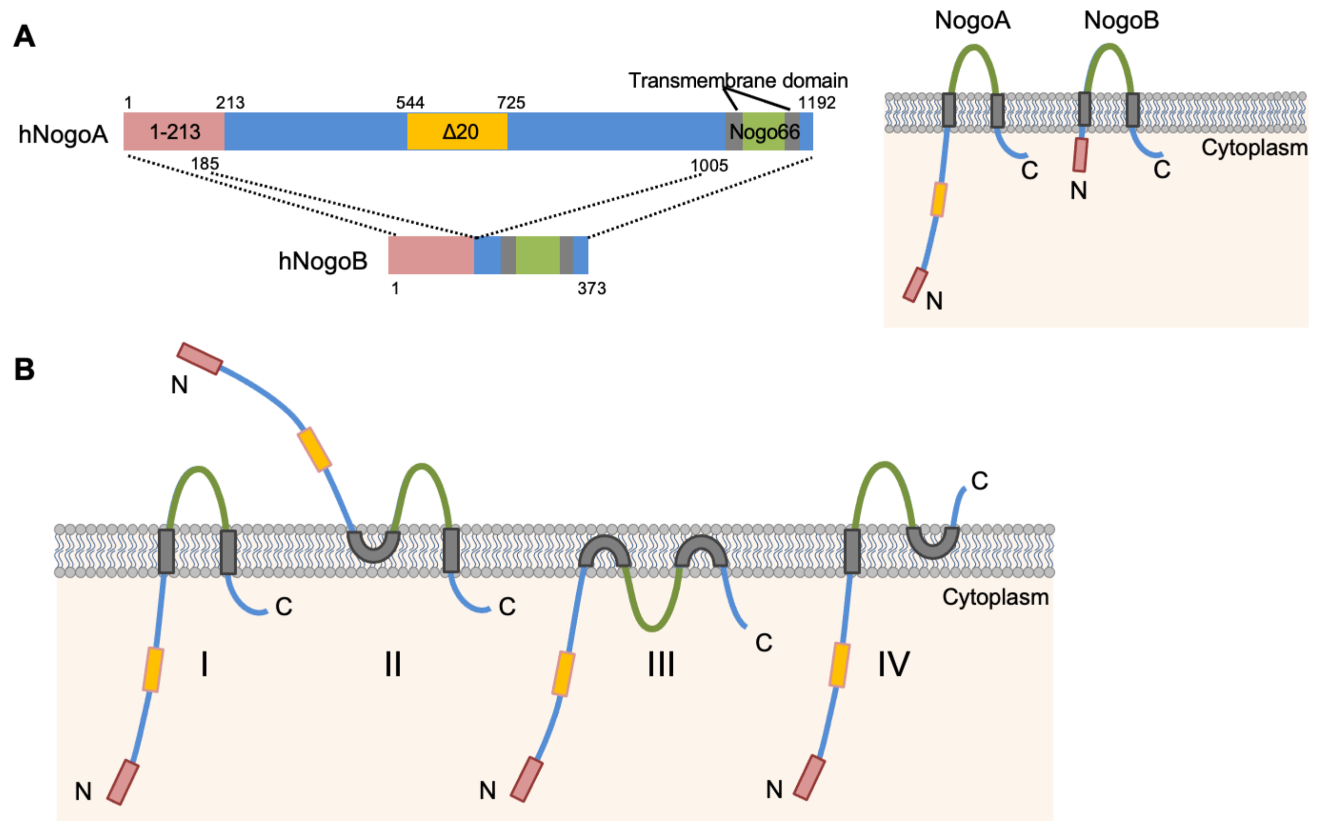

Supplementary Figure S1. Schematic illustrates of the NogoA and NogoB.
A, Domain structures of NogoA and NogoB. NogoA contains two growth inhibiting domains the Δ20 region and the Nogo-66 region.
B, The possible membrane topologies of NogoA[1] (I[2], II[3], III[4] and IV[5]).

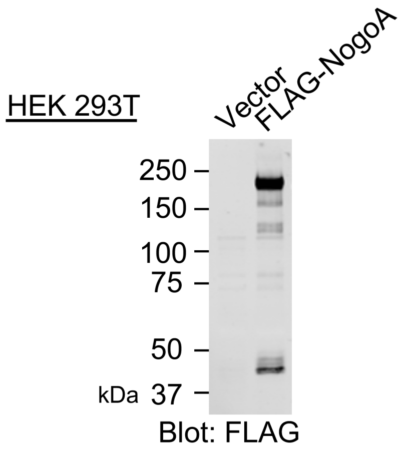

Supplementary Figure S2. Proteolysis of overexpressed NogoA.
HEK293T cells were transfected with Vector or FLAG-tagged NogoA and immunoblotted with anti-FLAG antibody.

```
                                                              hNogoA 1-213
Human 1     MEDLDQSPLVSSSDS-PPRPQPAFKYQFVREPEDEEEEEEEEEEDEDEDLEELEVLERKP  59  hNogoB
            MED+DQS LVSSS   PPRP PAFKYQFV EPEDEE+EE+EEEE++DEDLEELEVLERKP
Mouse 1     MEDIDQSSLVSSSADSPPRPPPAFKYQFVTEPEDEEDEEDEEEEEDDEDLEELEVLERKP  60  mNogoB

60          AAGLSAAPVPTAPAAGAPLMDFGNDFVPPAPRGPLPAAPPVAPERQPSWDPSPVSSTVPA  119
            AAGLSAA     P A APL+DF +D VPPAPRGPLPAAPP APERQPSW+ SP +S   A
61          AAGLSAA---PVPPAAAPLLDFSSDSVPPAPRGPLPAAPPTAPERQPSWERSPAAS---A  114

120         PSPLSAAAVSPSKLPEDDEPPARPPPPPPASVSPQAEPVWTPPAPAPAAPPSTPAAPKRR  179
            PS   AAAV PSKLPEDDE             +    P    P   PAAPPSTPAAPKRR
115         PSLPPAAAVLPSKLPEDDE-----------PPARPPAPAGASPLAEPAAPPSTPAAPKRR  163

180         GSSGSVDETLFALPAASEPVIRSSAEN-MDLKEQPGNTISAGQEDFPSVLLETAASLPSL  238
            GS GSVDETLFALPAASEPVI SSAE  MDLKEQPGNT+S+GQEDFPSVL ETAASLPSL
164         GS-GSVDETLFALPAASEPVIPSSAEKIMDLKEQPGNTVSSGQEDFPSVLFETAASLPSL  222

239         SPLSAASFKEHEYLGNLSTVLPTEGTLQENVSEASKEVSEKAKTLLIDRDLTEFSELEYS  298
            SPLS  SFKEH YLGNLS V  TEGT++E ++EAS+E+ E+A    ++R+  EFS LEYS
223         SPLSTVSFKEHGYLGNLSAVASTEGTIEETLNEASRELPERATNPFVNRESAEFSVLEYS  282

299         EMGSSFSVSPKAESAVIVANPREEIIVKNKDEEEKLVSNNILHNQQELPTALTKLVKEDE  358
            EMGSSF+ SPK ESA++V N +EE+IV++KD+E+ LV +  LHN QE P  LTK+VKED
283         EMGSSFNGSPKGESAMLVENTKEEVIVRSKDKED-LVCSAALHNPQESPATLTKVVKEDG  341

359         VVSSEKAKDSFNEKRVAVEAPMREEYADFKPFERVWEVKDSKEDS-DMLAAGGKIESNLE  417
            V+S EK  D FNE +++V AP+REEYADFKPFE+ WEVKD+ E S D+LAA     +N+E
342         VMSPEKTMDIFNEMKMSVVAPVREEYADFKPFEQAWEVKDTYEGSRDVLAA----RANME  397

418         SKVDKKCFADSLEQTNHEKDSESSNDDTSFPSTPEGIKDRSGAYITCAPFNPAATESIAT  477
            SKVDKKCF DSLEQ  H KDSES N++ SFP TPE +KD S AYITC  F+ +ATES A
398         SKVDKKCFEDSLEQKGHGKDSESRNENASFPRTPELVKDGSRAYITCDSFS-SATESTAA  456

478         NIFPLLGDPTSENKTDEKKIEEKKAQIVTEKNTSTKTSNPFLVAAQDSETDYVTTDNLTK  537
            NIFP+L D TSENKTDEKKIEE+KAQI+TEK TS KTSNPFLVA  DSE DYVTTDNL+K
457         NIFPVLEDHTSENKTDEKKIEERKAQIITEK-TSPKTSNPFLVAIHDSEADYVTTDNLSK  515

538         VTEEVVANMPEGLTPDLVQEACESELNEVTGTKIAYETKMDLVQTSEVMQESLYPAAQLC  597
            VTE VVA MPEGLTPDLVQEACESELNE TGTKIAYETK+DLVQTSE +QES+YP AQLC
516         VTEAVVATMPEGLTPDLVQEACESELNEATGTKIAYETKVDLVQTSEAIQESIYPTAQLC  575

598         PSFEESEATPSPVLPDIVMEAPLNSAVPSAGASVIQPSSSPLEASS-VNYESIKHEPENP  656
            PSFEE+EATPSPVLPDIVMEAPLNS +PS GASV QPS+SPLE  S V+Y+ IK EPENP
576         PSFEEAEATPSPVLPDIVMEAPLNSLLPSTGASVAQPSASPLEVPSPVSYDGIKLEPENP  635

657         PPYEEAMSVSLKKVSGIKEEIKEPENINAALQETEAPYISIACDLIKETKLSAEPAPDFS  716
            PPYEEAMSV+LK  S  KEEIKEPE+ NAA QE EAPYISIACDLIKETKLS EP+P+FS
636         PPYEEAMSVALK-TSDSKEEIKEPESFNAAAQEAEAPYISIACDLIKETKLSTEPSPEFS  694

717         DYSEMAKVEQPVPDHSELVEDSSPDSEPVDLFSDDSIPDVPQKQDETVMLVKESLTETSF  776
            +YSE+AK E+ VPDH ELV+DSSP+SEPVDLFSDDSIP+VPQ Q+E VML+KESLTE S
695         NYSEIAKFEKSVPDHCELVDDSSPESEPVDLFSDDSIPEVPQTQEEAVMLMKESLTEVS-  753

777         ESMIEYENKEKLSALPPEGGKPYLESFKLSLDNTKDTLLPDEVSTLSKKEKIPLQMEELS  836
            E++ ++++KE+LSA P E GKPYLESF+ +L  TKD    +E+ TL+KKE I LQMEE +
754         ETVTQHKHKERLSASPQEVGKPYLESFQPNLHITKDAA-SNEIPTLTKKETISLQMEEFN  812

837         TAVYSNDDLFISKEAQIRETETFSDSSPIEIIDEFPTLISSKTDSFSKLAREYTDLEVSH  896
            TA+YSNDDL  SKE +++E+ETFSDSSPIEIIDEFPT +S+K DS    +EYTDLEVS+
813         TAIYSNDDLLSSKEDKMKESETFSDSSPIEIIDEFPTFVSAKDDS----PKEYTDLEVSN  868

897         KSEIANAPDGAGSLPCTELPHDLSLKNIQPKVEEKISFSDDFSKNGSATSKVLLLPPDVS  956
            KSEIAN   GA SLPC+ELP DLS KN  PK E  +S  D+FSK+ S+ SKV LL P+VS
869         KSEIANVQSGANSLPCSELPCDLSFKNTYPKDEAHVS--DEFSKSRSSVSKVPLLLPNVS  926

957         ALATQAEIESIVKPKVLVKEAEKKLPSDTEKEDRSPSAIFSAELSKTSVVDLLYWRDIKK  1016
            AL +Q E+ +IVKPKVL KEAE+KLPSDTEKEDRS +A+ SAEL+KTSVVDLLYWRDIKK
927         ALESQIEMGNIVKPKVLTKEAEEKLPSDTEKEDRSLTAVLSAELNKTSVVDLLYWRDIKK  986

1017        TGVVFGASLFLLLSLTVFSIVSVTAYIALALLSVTISFRIYKGVIQAIQKSDEGHPFRAY  1076
            TGVVFGASLFLLLSLTVFSIVSVTAYIALALLSVTISFRIYKGVIQAIQKSDEGHPFRAY
987         TGVVFGASLFLLLSLTVFSIVSVTAYIALALLSVTISFRIYKGVIQAIQKSDEGHPFRAY  1046

1077        LESEVAISEELVQKYSNSALGHVNCTIKELRRLFLVDDLVDSLKFAVLMWVFTYVGALFN  1136
            LESEVAISEELVQKYSNSALGHVN TIKELRRLFLVDDLVDSLKFAVLMWVFTYVGALFN
1047        LESEVAISEELVQKYSNSALGHVNSTIKELRRLFLVDDLVDSLKFAVLMWVFTYVGALFN  1106

1137        GLTLLILALISLFSVPVIYERHQAQIDHYLGLANKNVKDAMAKIQAKIPGLKRKAE  1192  hNogoB&C
            GLTLLILALISLFS+PVIYERHQAQIDHYLGLANK+VKDAMAKIQAKIPGLKRKAE
1107        GLTLLILALISLFSIPVIYERHQAQIDHYLGLANKSVKDAMAKIQAKIPGLKRKAE  1162  mNogoB&C
```

Supplementary Figure S3. Comparison of human and mouse NogoA amino acid sequences. Human NogoA N-fragment (hNogoA 1-213) is boxed with blue. NogoB sequences for human (pink and orange) and mouse (purple and red) are indicated. Human NogoA N-fragment contains green colored sequence which is not contained in NogoB.

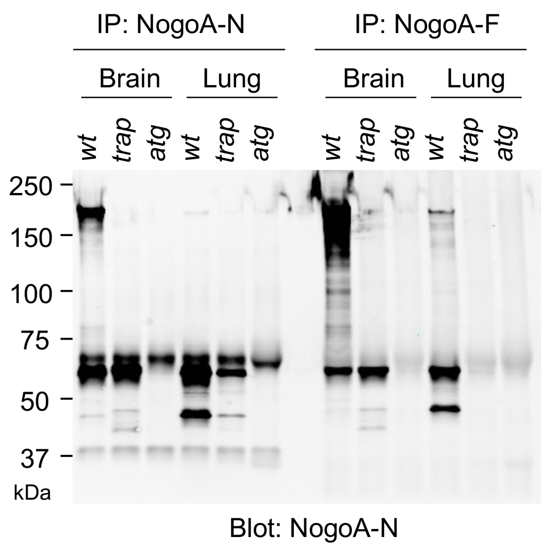

Supplementary Figure S4. Immunoprecipitation of the 40 kDa NogoA fragment using anti-NogoA-N and NogoA-F antibodies.
Mouse brains and Lungs from *wt*, $NogoA^{trap/trap}$, and $NogoA^{atg/atg}$ were lysed and immunoprecipitated with anti-NogoA-N and NogoA-F antibodies. The samples were blotted with anti-NogoA-N antibody.

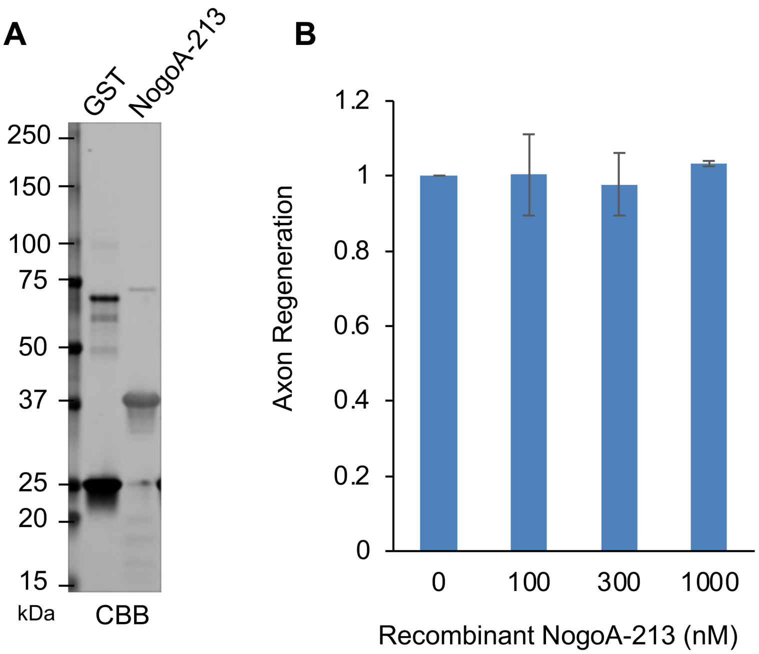

Supplementary Figure S5. Effect of recombinant NogoA N-fragment protein on axonal regeneration.
A, Recombinant proteins purified from E.Coli were resolved in SDS-PAGE and stained with CBB.
B, Cortical neurons were scraped at div 8 and allowed to regenerate for 3 days in the presence of indicated amount of recombinant NogoA 1-213. Quantification of axonal regeneration relative to control. Mean ± SE, n = 3 biological replicates.

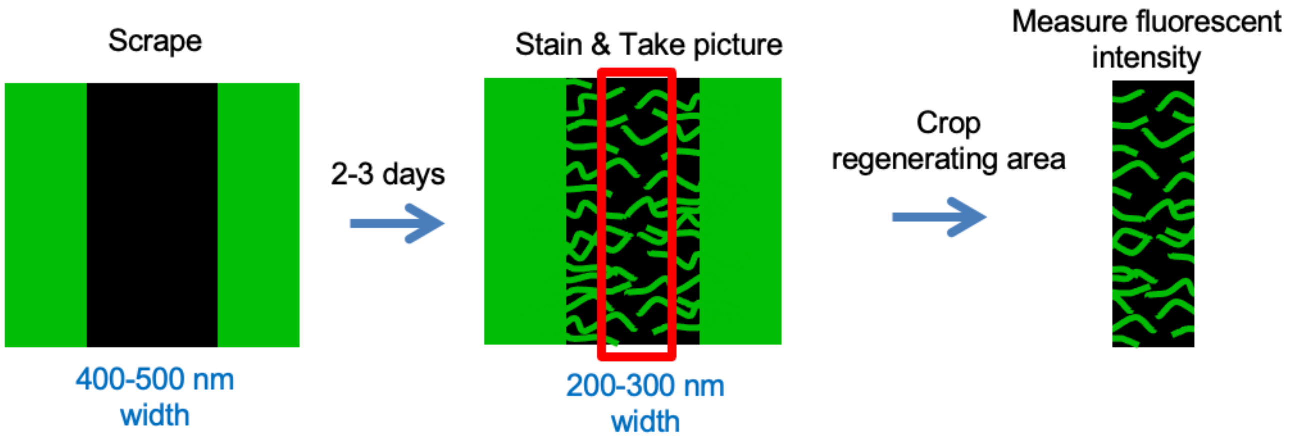

Supplementary Figure S6. Scheme of in vitro axon regeneration assay.
Neurons were cultured in a 96-well plate as a monolayer and scraped with a pin tool as axotomy. A couple of days after scraping, neurons were fixed and stained with ßIII-Tubulin antiboty and taken photos. Scraped area was cropped and the fluorescent intensity was measured as regenerating axons.

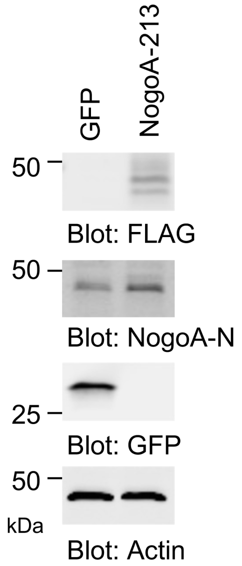

Supplementary Figure S7. Expression of transfected NogoA N-terminal fragment in cortical culture neurons.
Cortical neurons transfected with GFP or FLAG-NogoA 1-213 were cultured for 11 days and then lysed and immunoblotted with indicated antibodies.

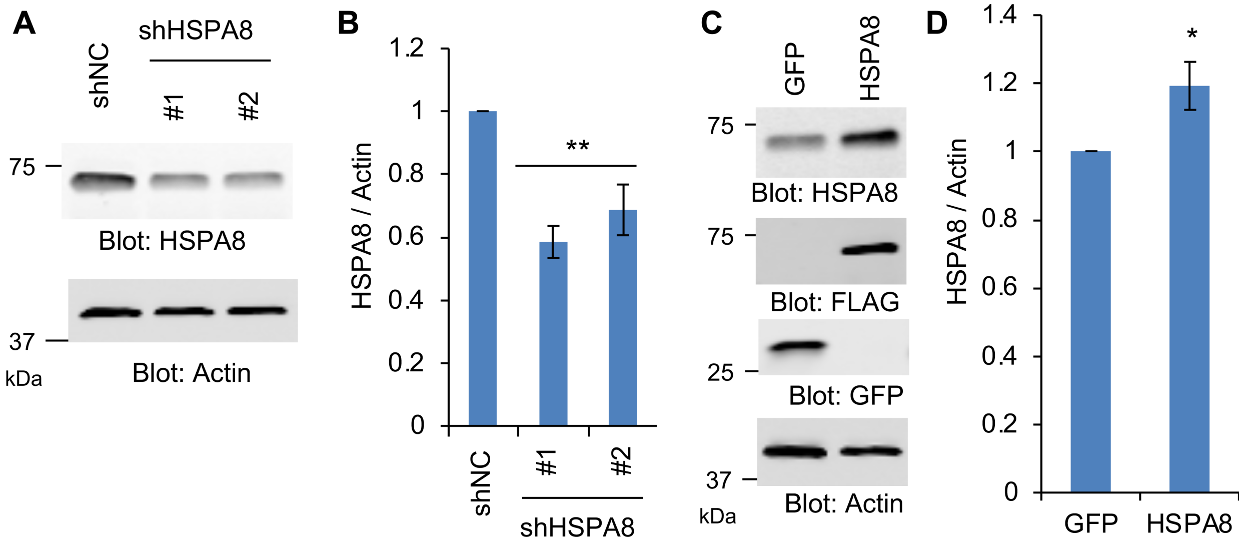

Supplementary Figure S8. Expression of HSPA8 in knocked-down or over-expressed cortical culture neurons.

A, Cortical neurons transfected with shNC, shHSPA8 #1 or #2 were cultured for 11 days and then lysed and immunoblotted with anti-HSPA8 and anti-Actin antibodies.

B, Quantification of HSPA8 protein levels divided by Actin normalized to shNC. Mean ±SE, N=4 independent experiments. **$p<0.01$, one-way ANOVA followed by Dunnett's test.

C, Cortical neurons transfected with GFP or FLAG-HSPA8 were cultured for 11 days and then lysed and immunoblotted with indicated antibodies.

D, Quantification of HSPA8 protein levels divided by Actin normalized to GFP. Mean ±SE, N=4 independent experiments. *$p < 0.05$, Student's two-tailed *t* test.

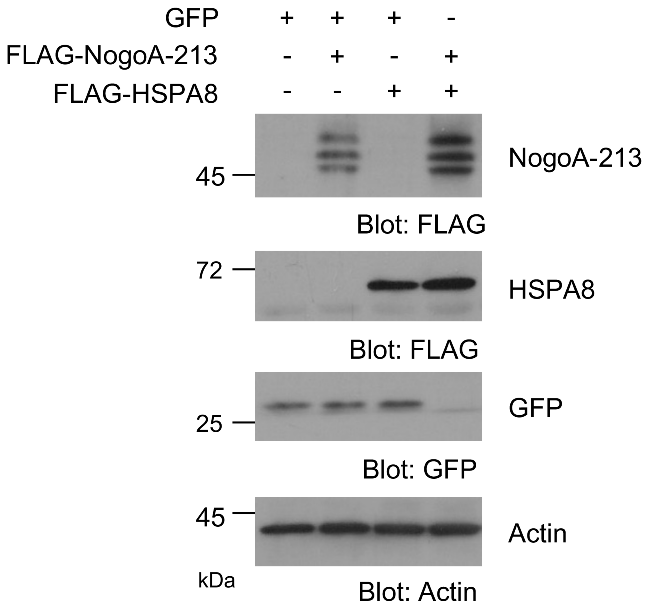

Supplementary Figure S9. Expression of transfected NogoA-213 and HSPA8 in cortical culture neurons.

Cortical neurons transfected with GFP, FLAG-NogoA-213, FLAG-HSPA8, or FLAG-NogoA-213+ FLAG-HSPA8 were cultured for 11 days and then lysed and immunoblotted with indicated antibodies.

References

1. Teng, F.Y. and B.L. Tang, *Cell autonomous function of Nogo and reticulons: The emerging story at the endoplasmic reticulum.* J Cell Physiol, 2008. **216**(2): p. 303-8.
2. GrandPré, T., et al., *Identification of the Nogo inhibitor of axon regeneration as a Reticulon protein.* Nature, 2000. **403**(6768): p. 439-44.
3. Hu, F., et al., *Nogo-A interacts with the Nogo-66 receptor through multiple sites to create an isoform-selective subnanomolar agonist.* J Neurosci, 2005. **25**(22): p. 5298-304.
4. Voeltz, G.K., et al., *A class of membrane proteins shaping the tubular endoplasmic reticulum.* Cell, 2006. **124**(3): p. 573-86.
5. Sekine, Y., J.A. Lindborg, and S.M. Strittmatter, *A proteolytic C-terminal fragment of Nogo-A (reticulon-4A) is released in exosomes and potently inhibits axon regeneration.* J Biol Chem, 2020. **295**(8): p. 2175-2183.
